# Recent Advances in Measurement and Dietary Mitigation of Enteric Methane Emissions in Ruminants

**DOI:** 10.3389/fvets.2016.00039

**Published:** 2016-05-20

**Authors:** Amlan K. Patra

**Affiliations:** ^1^Department of Animal Nutrition, Faculty of Veterinary and Animal Sciences, West Bengal University of Animal and Fishery Sciences, Kolkata, India

**Keywords:** methane, ruminants, measurement method, mitigation technology, diet

## Abstract

Methane (CH_4_) emission, which is mainly produced during normal fermentation of feeds by the rumen microorganisms, represents a major contributor to the greenhouse gas (GHG) emissions. Several enteric CH_4_ mitigation technologies have been explored recently. A number of new techniques have also been developed and existing techniques have been improved in order to evaluate CH_4_ mitigation technologies and prepare an inventory of GHG emissions precisely. The aim of this review is to discuss different CH_4_ measuring and mitigation technologies, which have been recently developed. Respiration chamber technique is still considered as a gold standard technique due to its greater precision and reproducibility in CH_4_ measurements. With the adoption of recent recommendations for improving the technique, the SF_6_ method can be used with a high level of precision similar to the chamber technique. Short-term measurement techniques of CH_4_ measurements generally invite considerable within- and between-animal variations. Among the short-term measuring techniques, Greenfeed and methane hood systems are likely more suitable for evaluation of CH_4_ mitigation studies, if measurements could be obtained at different times of the day relative to the diurnal cycle of the CH_4_ production. Carbon dioxide and CH_4_ ratio, sniffer, and other short-term breath analysis techniques are more suitable for on farm screening of large number of animals to generate the data of low CH_4_-producing animals for genetic selection purposes. Different indirect measuring techniques are also investigated in recent years. Several new dietary CH_4_ mitigation technologies have been explored, but only a few of them are practical and cost-effective. Future research should be directed toward both the medium- and long-term mitigation strategies, which could be utilized on farms to accomplish substantial reductions of CH_4_ emissions and to profitably reduce carbon footprint of livestock production systems. This review presents recent developments and critical analysis on different measurements and dietary mitigation of enteric CH_4_ emissions technologies.

## Introduction

Greenhouse gas (GHG) emissions, largely methane (CH_4_) from the rumen and nitrous oxide from manure management, from livestock contribute considerably to the atmospheric GHG (1, 2). CH_4_ is normally produced during microbial fermentation of feeds, mainly structural carbohydrates, in the rumen by methanogenic archaea. Globally, about 95 million tones of CH_4_ are emitted from enteric fermentation of domestic animals in 2010 with an annual growth rate of 0.90% (2). Enteric CH_4_ contributes 17 and 3.3% of global CH_4_ and GHG emissions, respectively, which mostly arises from ruminant livestock (3). The contribution of GHG from livestock is expected to grow due to increasing populations of livestock animals triggered by an increasing demand of animal protein, especially in developing countries. Abatement of enteric CH_4_ emission is required to minimize the liability of livestock production for GHG emission. Mitigation strategies of enteric CH_4_ are considered to be less expensive than carbon dioxide (CO_2_) emissions (3, 4). Inhibition of CH_4_ emission by some technologies generally does not cause much detrimental effects on rumen fermentation, but may improve rumen fermentation efficiency. Sometimes, CH_4_ mitigation options are associated with improved efficiency of animal production, which is advantageous both environmentally and nutritionally. Several comprehensive reviews have been published recently, which describe a number of options and strategies to mitigate GHG from livestock production (3, 5–7). Generally, these review papers on CH_4_ mitigation technologies have been focused on research conducted during the past 10–15 years.

An accurate, cost-effective and repeatable measurement technique of enteric CH_4_ production from ruminants is required to evaluate a CH_4_ mitigation technology, preparation of inventory of CH_4_ gas emissions and assessment of carbon footprint of livestock products. Therefore, it has been an important area of research to develop new techniques, to improve accuracy of the existing methods and to compare among the methods of CH_4_ measurements in recent years. A number of methods are used to measure CH_4_ emission from ruminants, all of which differ in their application, cost, accuracy, precision, and repeatability depending on their conditions of use [e.g., Ref. (8–11)]. This paper primarily presents the latest advances in measurements and dietary mitigations of CH_4_ emissions from ruminants.

## Measurement Methods of CH_4_ Emissions

**Figure 1:**
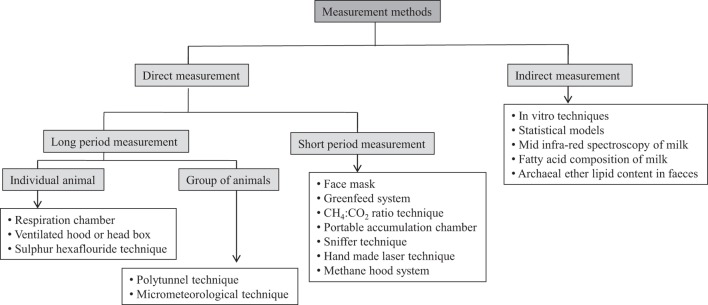
**A schematic presentation of different CH_4_ measurement techniques in ruminants using different approaches**.

A number of methods have been developed and improved in recent years, which is employed for long-term and short-term measurements in individual animals or grouped animals directly as well as indirect prediction of CH_4_ emissions (Figure 1). These methods are described here along with their advantages and disadvantages.

### Long-Term Measurement Methods

#### Respiratory Chamber Technique

Respiration chamber (RC) technique was used for determining energy balance and gaseous exchange in animals for many years [e.g., Ref. (12, 13)]. The principle of this technique is to measure the concentrations of CH_4_ (coming out through all avenues, i.e., mouth, nostrils, and rectum from enteric fermentation) in gas samples and total volume of air removed from the RC (10). An air pump continuously removes air from a RC through a flow meter in the open-circuit system to calculate volume of air removed. Outlet gas from the RC is continuously sampled for analysis through a duct system. The RC system is equipped with ventilation fans inside the chamber for proper mixing of expired gases and incoming air. Fresh air to the RC is directly drawn from outside or through an air conditioning system to control humidity and temperature. The RC is fitted with humidity, temperature, and barometric pressure meters to determine gas volume at standard temperature and pressure (STP) conditions (9).

The RC method for measurement of enteric CH_4_ production is often considered as the “gold standard” technique due to high accuracy and repeatability, and low animal-to-animal variations of CH_4_ measurement using this technique (14, 15). However, RC system is costly for establishment and labor intensive for operation. This method imposes restrictions on eating and other natural behaviors of the animals resulting in CH_4_ production that often differs from CH_4_ production in their normal environments. This technique also requires great technical expertise to generate accurate CH_4_ emission measurements. Their potential negative effects on feed intake and milk production of lactating animals while confined in the RC can be minimized with proper adaptation of the animals to the system, but must also be considered. Moreover, RC method has limited “throughput,” and thus is less suitable when CH_4_ measurements on large numbers of animals are required, such as screening of low CH_4_-producing animals for genetic selection.

The RC technique can be highly accurate and precise when used with rigor and it measures total CH_4_ emission, including losses from anus and rumen fistulas. This method has additional advantage of measurement of gas production or consumption of other gases (e.g., oxygen, CO_2_, hydrogen, ammonia). In RC, the design may also allow to investigate other nutritional evaluations, such as digestibility, nitrogen balance, and energy metabolism. The RC method can detect relatively small effects of diets and supplements on CH_4_ emission determined on a small number of animals (9). With repeated measurements over the course of daily CH_4_ production patterns, RC technique can be employed to characterize diurnal CH_4_ emission variations, which may provide insight into underlying mechanisms of enteric CH_4_ formation, including relationships with H_2_ production when anti-methanogenic compounds are used. Despite the better accuracy and many nutritional advantages in the RC method, this method could invite significant errors in CH_4_ measurement unless proper calibration procedures are regularly followed. For example, in a recent ring test of calibration of RC in different laboratories, ducting system resulted in 15.3% variations, followed by errors in mixing of air in the RC by 3.4% and analyzer by 1.3% variations (16). In conclusion, although RC technique is considered as “gold standard” in determining CH_4_ emission due to high accuracy and low animal and day variation of measurements, low throughput, high cost, imposing restriction on natural animal behavior, and greater technical requirements for operation limit this technique for widespread use.

#### Sulfur Hexafluoride Tracer Technique

The sulfur hexafluoride (SF_6_) tracer technique was developed by Zimmerman (17) and was first used for measurement of CH_4_ in grazing cattle by Johnson et al. (18). The SF_6_ method has been used extensively during the last two decades for measurement of CH_4_ emissions from ruminants. The principle behind this method is that CH_4_ production can be measured if SF_6_ gas production rate from the rumen is known (18). Small permeation tubes are filled with SF_6_ and are then placed into the rumen of animals. Test animals are fitted with gas sampling apparatus, which consists of a halter to support capillary tubing whose inlets to be placed close to the nose and an evacuated canister to collect gas samples (10). Representative gas samples containing respired and eructated gas are collected usually for 24 h through capillary tubing connected to an evacuated canister. The tubing regulates the gas sampling rate (19). The concentrations of SF_6_ and CH_4_ in the gas samples collected in canister are analyzed by gas chromatography. The CH_4_ emission is calculated from the SF_6_ release rate and concentrations of SF_6_ and CH_4_ in the canister gas samples in excess of background concentrations in the air using the following equation (18).

$${\text{CH}}_{4}(\text{g}/\text{day})={\text{SF}}_{6}(\text{g}/\text{day})\times \left({\left[{\text{CH}}_{4}\right]}_{\text{c}}-{\left[{\text{CH}}_{4}\right]}_{\text{b}}\right)/\left({\left[{\text{SF}}_{6}\right]}_{\text{c}}-{\left[{\text{SF}}_{6}\right]}_{\text{b}}\right)$$

where [CH_4_]_c_ and [SF_6_]_c_ are the concentrations of CH_4_ and SF_6_ in the canister, respectively; while [CH_4_]_b_ and [SF_6_]_b_ are the CH_4_ and SF_6_ concentrations in the background air, respectively.

Unlike the RC method, the SF_6_ method can be employed on large numbers of animals concurrently to measure CH_4_ emissions from both grazing and non-grazing animals in comparatively less time and cost. Also, SF_6_ method can impose lesser effect on animal behaviors under typical animal management conditions (18). However, it is labor intensive and requires great technical expertise to minimize experimental errors of measuring CH_4_. There are concerns over the variability and repeatability of measurements (9, 20). In addition, the long-term instability of release rate of the permeation tubes remains a concern for use in studies with long period of experiments (21, 22), and background atmospheric gas concentrations can impact markedly on the success of the SF_6_ technique (23). Thus, the day-to-day and animal-to-animal variations in CH_4_ emission estimates are greater with the SF_6_ technique compared with the estimates derived from RC (20, 24, 25). These greater variations would induce a negative impact on the power of statistical analyses using this technique as it will require more animals (replicates) to detect treatment differences in evaluation of CH_4_ mitigation studies (9). The CH_4_ emissions measured by SF_6_ technique were similar to the values quantified by RC and ventilated head hood systems in some studies (14, 18, 26), but were different in other studies (14, 25, 27). Johnson et al. (18) observed that CH_4_ emission using the SF_6_ technique agreed with CH_4_ emission using RC technique in grazing cattle (7.3 versus 7.2% of gross energy intake). In subsequent studies (28, 29), slightly lower emissions (5–10%) were observed with the SF_6_ method than with the RC method for both cattle and sheep, which can partly be due to the few percent of CH_4_ lost via rectum. By contrast, higher values of CH_4_ emission were observed using the SF_6_ technique than RC technique (20, 27, 30). Muñoz et al. (25) also noted significantly higher mean values of CH_4_ emissions (gram/day) and CH_4_ yield (gram/kilogram DM intake) for the SF_6_ technique than for the RC method (443 versus 396 g/day and 26.7 versus 24.2 g/kg DM intake). Although average CH_4_ emissions may not be different in some studies, within- and between-animal coefficients of variations were much greater for the SF_6_ technique than for the RC method (14, 20, 31). Pinares-Patiño et al. (20) utilized the same animals for measurement of CH_4_ using with the SF_6_ and RC technique and reported that the within coefficients of variations were 4.7, 13.5, and 11.7% in RC technique, SF_6_ technique, and with SF_6_ technique performed inside RC, respectively. The between-animal variations were also considerably higher with the SF_6_ technique than with the RC technique.

A number of factors, such as release rate of SF_6_ from permeation tubes, influence of SF_6_ release rate on CH_4_ emission rate, measurements of background concentrations of SF_6_, flow rate in the tubing during sample gas collection, inconsistency of CH_4_ measurements using RC and SF_6_ techniques, and within- and between-animal variations, have been implicated for low accuracy in CH_4_ emission measurements in the SF_6_ technique (9, 22, 32). A considerable research effort has been undertaken to improve the accuracy, precision, and consistency of the results in the SF_6_ technique in different studies [e.g., Ref. (22, 23, 33)]. The determination of SF_6_ release rate over time from tubes is important, which affects emission estimates (19, 34). Permeation tubes that release SF_6_ in greater rates may result in higher CH_4_ production compared with the tubes with lower release rates (34). Hence, permeation tubes with similar SF_6_ release rate are recommended to obtain better accuracy, especially, for the comparison of different mitigation treatments (34). Permeation tubes are regularly weighed over 1 month under laboratory conditions to obtain the release rates of SF_6_ (9). Only highly linear permeation tubes are used for measuring experiments (19). The SF_6_ release rates from permeation tubes are calculated applying zero-order kinetics (19). However, Lassey et al. (19) demonstrated that the SF_6_ release rates from permeation tubes kept at 39°C in a dry laboratory incubator decreased with time. Thus, the application of zero-order kinetics to predict the SF_6_ release rate from permeation tubes invites a considerable error in measurement of enteric CH_4_ outputs, particularly when an experiment continues more than 30 days after the calibration of permeation tubes (22, 35). The inconsistency in measurements of CH_4_ emissions using the RC and SF_6_ techniques increased substantially when the permeation tubes were kept in the rumen for long time (21). Researchers have shown that the curves of the rate of SF_6_ release from permeation tubes are curvilinear under laboratory conditions (22, 27). Therefore, Lassey et al. (19) suggested the use of quadratic equations to predict the rate of SF_6_ release from permeation tubes with time. This approach was suggested to be certainly much better than using zero-order kinetics (22). Recently, Moate et al. (22) demonstrated that the use of Michaelis–Menten kinetics can more accurately describe the SF_6_ release rate pattern from permeation tubes and this may improve the accuracy of the measurement of CH_4_ emissions from ruminants. Using this kinetics, measurement of CH_4_ emissions can be continued for even up to 800 days after insertion of the tubes into the rumen compared with the typical period of 60–90 days. Another important factor is the design of capillary-tube flow rate restrictors that can also cause appreciable errors in measurement of CH_4_ emissions of up to 15.6% (33). Orifice plate flow restrictors can control the gas sample collection rate into canisters and lower errors in measurement of CH_4_ production (33). The SF_6_ technique using these modifications resulted in measurements of CH_4_ emission to be greatly accurate with measurements taken using RC (33). In this experiment, mean CH_4_ yield (gram/kilogram DM intake) was 21.9 ± 1.65 and 22.3 ± 1.44 in lactating dairy cows when measured by the RC and SF_6_ technique, respectively; and the between-animal coefficients of variation were 7.5 and 6.5% using the RC and SF_6_ technique, respectively.

The background SF_6_ and CH_4_ concentrations should be corrected, but determination of representative background concentrations of these gases under field conditions may be difficult because direction and velocity of wind and other animals in the vicinity may influence the background concentrations (23). Besides, the SF_6_ release from permeation tubes may affect the background concentration of SF_6_ to which an adjacent animal is exposed (23). For this reason, Williams et al. (23) emphasized that recording of improper background concentrations of SF_6_ can affect the extent of CH_4_ emission measurements. From these observations, Lassey (36) concluded that the relationship between the estimated CH_4_ emission rates and the SF_6_ release rates from permeation tubes was merely an artifact as a result of inappropriate background SF_6_ concentrations. Pinares-Patiño et al. (34) suggested that this concern could be ameliorated to a certain degree by using all permeation tubes with similar rate of SF_6_ release in an experiment. In summary, the SF_6_ method can be employed without imposing much effect on natural behavior of grazing animals and has a greater throughput, but it could result in greater variations in CH_4_ emissions compared with the RC method. Nevertheless, the SF_6_ method can be used with a high level of precision when recent recommendations of this technique are followed for measurement of CH_4_ emissions.

#### Ventilated Hood Chambers or Head Box System

This system also can be used for measurement of daily CH_4_ emissions using the same principles as the RC method [e.g., Ref. (37, 38)]. In this technique, a box or hood is fabricated to accommodate the head of animals, and air samples drawn through the hoods are analyzed for CH_4_ concentrations in incoming and exhaust air (8). Unlike RC, this method does not quantify CH_4_ arising from the hindgut. Airflow is measured and used to calculate CH_4_ emission. Head chambers are typically large enough to allow the animal to move its head in an unrestricted manner and obtain feed and water. Like RC, they can be used to obtain continuous measurements over successive 24-h periods. Ventilated head hoods with different designs and sizes for small and large animals have been installed in different countries for nutritional studies [e.g., Ref. (37–40)]. The flow rate of exhaust air is important for the accuracy of gas analysis and comfort of the animals in head box (37). This system could also be employed for nutritional evaluation and energy metabolism of feeds [e.g., Ref. (40)]. Boadi et al. (26) compared ventilated head hood with the SF_6_ technique. The average daily CH_4_ production was similar for both the methods (130 and 137 L/day for head hood and SF_6_ method, respectively). Animal-to-animal variation was, however, significant with the SF_6_ method (11.7%), but not with the head hood technique (0.1%) for production. Troy et al. (41) obtained simultaneous measurements of CH_4_ outputs from cattle using RC and feeder-mounted hoods located within RC. It was reported that increases in concentrations of CH_4_ in hoods over RC background were positively correlated (*r* = 0.67) with daily CH_4_ emissions, but there was substantial variability. This technique measures CH_4_ emissions reliably similar to the RC method and involves low cost compared with RC, but animals are required an extensive training to become adapted to the head hood, which restricts its extensive use for screening of large numbers of animals for genetic selection purpose.

### Short-Term Measurement Methods

#### Face Mask Method

Face-masks for “spot-sampling” of respiratory exchange and CH_4_ emission have been used in cattle, sheep, and goats for many years [e.g., Ref. (42)]. In this method, animals are needed to train to stay in sternal recumbency for the measurement periods (e.g., 30 min) repeated over the course of 24-h periods (8). This method presents a greater animal and day variation and only provides a short-term emission rate (43). The CH_4_ emission values using this technique are highly dependent upon the number and timing of respiratory exchange measurements taken with respect to diurnal patterns of feeding cycle and CH_4_ emission. However, if sufficient data are collected from several animals with greater regularity in sample collection throughout the measurement period of 24 h, a typical CH_4_ emission pattern can be calculated (11). Face mask can be useful for short-term measurements of CH_4_ emission rate for screening of large numbers of animals, but may cause marked discomfort and distress and change behaviors of the animals, and consequently, can affect the gas measurements.

#### Portable Accumulation Chamber

Portable accumulation chamber (PAC) system is essentially a RC without airflow. In this technique, PAC acts to trap all exhaled gases (CH_4_, CO_2_, and other gases), while oxygen depletes during the collection period of 1–2 h, and a single CH_4_ or other gas measurement is taken at the end of the collection period (44, 45). Emission of CH_4_ is calculated as the concentration of CH_4_ (corrected for background) multiplied by net chamber volume, adjusted for STP, divided by time of measurement (44). The time period of use should be restricted to avoid negative effects of increased chamber CO_2_ concentration, and accordingly the PAC is essentially a short-term respiration measurement. Moderate repeatability (correlation of 0.33–0.43) of measurements of CH_4_ emission by individual sheep using PAC was reported in studies at different sites (46). This technique could be useful for screening of low CH_4_-producing animals from large herds for genetic improvement purpose.

#### CH_4_/CO_2_ Ratio Technique

The CH_4_:CO_2_ ratio method, which was conceptualized by Madsen et al. (47), determines CH_4_ emission from individual animals based on the calculated CO_2_ emission and CH_4_ and CO_2_ concentrations measured using a gas analyzer. Their method relies on analyzing air samples for CH_4_ and CO_2_ simultaneously with a gas analyzer that is based on Fourier transform infrared (FTIR) detection, and uses CO_2_ from the breath of animals as the tracer gas. Emission of CO_2_ can be predicted based on estimates of energy metabolism, heat production, and respiratory quotient (RQ), or carbon balance (47).

$${\text{CH}}_{4}(\text{g}/\text{d})={\text{CO}}_{2}\times (\text{g}/\text{day})\times \left({\left[{\text{CH}}_{4}\right]}_{\text{BS}}-{\left[{\text{CH}}_{4}\right]}_{\text{BG}}\right)/\left({\left[{\text{CO}}_{2}\right]}_{\text{BS}}-{\left[{\text{CO}}_{2}\right]}_{\text{BG}}\right)$$

where [CH_4_]_BS_ and [CO_2_]_BS_ are the CH_4_ and CO_2_ concentrations in the breath samples, respectively; while [CH_4_]_BG_ and [CO_2_]_BG_ are the CH_4_ and CO_2_ concentrations in the background air, respectively.

The repeatability of the CH_4_:CO_2_ ratio was reported to be 0.39 for Holsteins and 0.34 for Jerseys (48). Hellwing et al. (49) compared predicted CH_4_ emissions in lactating dairy cows using the CH_4_:CO_2_ ratio technique with CH_4_ emissions in the RC method and reported a positive relationship (*r* = 0.55) between the two methods, but the CH_4_:CO_2_ ratio technique significantly underestimated the CH_4_ production (412 versus 345 g/day). This difference may be due to an error in the prediction of within-day variation in CO_2_ emission, which needs to be improved to obtain better individual animal CH_4_ emission estimates (49). Several factors, such as diurnal variations in the CH_4_:CO_2_ ratio resulted from differences in digestive and metabolic activity and rumen fermentation pattern associated with feed intake and feeding frequency, and source of gas samples (e.g., exhaled air, flatus and fermentation of manure or bedding) could affect CH_4_ emission measurement (47, 50). Therefore, adequate numbers of measurements in different times of the days should be considered to account for diurnal and postprandial variation in CH_4_ and CO_2_ emissions in animals. Nonetheless, this technique could be employed to generate large-scale data for genetic evaluation of CH_4_ production.

#### GreenFeed System

GreenFeed (GF) system (C-lock Inc., Rapid City, SD, USA) has recently been patented by Zimmerman (51) for measurement of CH_4_, CO_2_, and H_2_ production from animals. This system includes an automatic baiting system, measurements of air flow and gas concentration systems, electronics and communication devices, a gas tracer device, and an animal detection system during visit of an animal to the unit. A detailed description and visualization of the system is provided in Hristov et al. (52). In GF system, CH_4_ emission is measured for a short period when animals visit to the system to consume feeds that are used as enticement. The concept of the GF system is that numerous short-term CH_4_ emission values from an individual animal measured in different times within a day for many days can be aggregated to estimate an average daily CH_4_ emission from the animal. Software function allows investigators to control the timing of feed availability and to allocate CH_4_ measurements across various times of the day. The animals entering an automatic feeding system are recognized and concentrations of CH_4_ are measured at that particular time. Air is constantly pumped out through the automatic feeding system to measure flow rate and thereby CH_4_ emission during feeding period. Daily CH_4_ emission is calculated using the same principle as in RC method, whereby CH_4_ emission rate is calculated using volumetric air flow rate adjusted to STP and corrected for capture rate, as detailed by Huhtanen et al. (50):

$${\text{CH}}_{4}(\text{L}/\text{min})={\text{C}}_{\text{p(i)}}\times \left({\left[{\text{CH}}_{4}\right]}_{\text{c(i)}}-{\left[{\text{CH}}_{4}\right]}_{\text{b(i)}}\right)\times {\text{F}}_{\text{air(i)}}/10^{6}$$

where C_p(i)_ is the fractional capture rate of air at time i; [CH_4_]_c(i)_ and [CH_4_]_b(i)_ are the concentrations of captured gas (ppm) and background gas of CH_4_ (ppm), respectively, time i; and F_air(i)_ is the volumetric air flow rate (L/min) measured on a dry-gas basis at time i.

The GF system was compared with the RC and SF_6_ techniques to assess the accuracy and suitability of the GF system for measurement and detection of treatment difference for CH_4_ emissions in different studies (53, 54). The mean value of CH_4_ emission by growing dairy cattle in GF system was similar to measurements taken in the RC system, but was lower than the values obtained using the SF_6_ technique (53). Dorich et al. (54) noted that the average CH_4_ production from lactating dairy cows measured using the GF and SF_6_ techniques were similar (468 versus 467 g/day), but the GF method resulted in smaller coefficients of variation (14.1–22.4 versus 16.0–111%) for CH_4_ emissions, and higher relationship (0.65 versus 0.41) between CH_4_ (gram/day) and dry matter (DM) intake compared with GF system. The authors attributed this higher variability for SF_6_ measurements to the high concentration of background gases combined with poor barn ventilation. Also, Arbre et al. (55) analyzed repeatability estimates and noted that 3-day periods were necessary for the SF_6_ technique and 17-day periods for the GF system to achieve the repeatability of 0.70 for CH_4_ yield (gram/kilogram DM intake). The repeatability did not increase after 4-day periods for the SF_6_ method (repeatability = 0.73), but increased for the GF method until 45-day periods (repeatability = 0.90). Hammond et al. (53) conducted three experiments (two experiments on indoor animals and one experiment on grazing animals) in which Holstein heifers were fed various diets to compare the GF system with the RC or SF_6_ method. Daily CH_4_ emissions (gram/day) and CH_4_ yield (gram/kilogram DM intake) were similar between the GF (198 g/day and 26.6 g/kg DM intake in experiment 1; and 208 g/day and 27.8 g/kg DM intake in experiment 2) and RC (218 g/day and 28.3 g/kg DM intake in experiment 1; and 209 g/day and 27.7 g/kg DM intake in experiment 2) methods in both indoor experiments. In experiment 3, CH_4_ emissions and yields determined using the SF_6_ technique were, however, greater than the values measured using the GF system during grazing (186 versus 164 g/day and 21.6 versus 18.8 g/kg DM intake). Moreover, CH_4_ production quantified by the GF technique was not concordant (*r* = 0.10) with CH_4_ production determined by the RC method, but was only in moderate agreement (*r* = 0.60) with CH_4_ production measured by the SF_6_ technique. Significant treatment and individual animal differences in CH_4_ emission were detected using both RC and SF_6_ techniques, but were unable to detect using the GF method (53). This was attributed to a limited number of measurements obtained with the GF system in grazing animals and the timing of the measurements relative to daily patterns of CH_4_ emission, highlighting the importance of obtaining sufficient numbers of observations using the GF system. However, Velazco et al. (56) reported that GF and RC methods produced similar CH_4_ emissions (209.7 versus 215.1 g CH_4_/day) and also CH_4_ yield (22.7 versus 23.7 g CH_4_/kg of DM intake).

In principle, this technique requires sufficient numbers of measurements over time to obtain accurate estimates of daily emission, and relies on animals voluntarily visiting the unit (56). Some animals may not visit the GF unit for sufficient times despite feed restriction (57). Compared with the RC and SF_6_ techniques, GF system requires more time and animals, when a study is planned for comparison of CH_4_ production among the treatments, due to higher within-day and within-animal variance (58). The use of GF system requires a feed supplement, which may also introduce between-day variation with respect to supplement consumption and interact with the actual treatments. Taken together, CH_4_ emissions quantified using GF method may show high variability compared with the emissions measured by the SF_6_ and RC methods; the GF method, however, offers a lower cost alternative as an automated method for measurement of CH_4_ emissions from individual animal than SF_6_ and RC methods both in indoor and grazing conditions.

#### Sniffer Method

This technique was first conceptualized by Garnsworthy et al. (59). In this technique, a sampling inlet is placed in the feed manger of an automatic milking system to collect air eructed by cattle during milking (often called the “sniffer” technique). As described by Garnsworthy et al. (59), air in the manger is constantly sampled, analyzed, and logged at 1-s intervals using data loggers to measure CH_4_ and CO_2_ concentrations in close proximity to the muzzle of the animal. Information on eructation frequency and CH_4_ released per eructation are used to estimate CH_4_ emission rate by individual animal during milking. Garnsworthy et al. (59) reported a good relationship (*r* = 0.79) between the measurements of CH_4_ emission using the RC technique and CH_4_ emission rate using this method. By contrast, Huhtanen et al. (50) compared the measurements of eructated CH_4_ concentration with CH_4_ emissions determined using the GF system in lactating dairy cows in two experiments. They found between-cow coefficient of variation (11.0–17.6 versus 17.5–28.0%) was smaller for the GF system compared with the sniffer method. There was weak relationship (*R*^2^ = 0.09) between the CH_4_ measurements (gram/day) using GF system and concentrations of CH_4_ recorded by the sniffer method, which may be attributed to the inconsistent air-mixing conditions within the feeding troughs influenced by the geometry of feed troughs, muzzle movement, and muzzle position (50). Thus, further research is needed if this type of sniffer method could be employed for quantification of CH_4_ with some consistency.

#### Hand Laser CH_4_ Detector

The hand laser CH_4_ detector (LMD) technique (60, 61) measures exhaled CH_4_ concentrations in the air near the nose or mouth of an animal in normal environment. The data consist of a series of peaks representing the animal’s respiratory cycle. Only peaks reflecting the increase in CH_4_ concentrations due to exhalation or eructation are used in the analysis (61). As the measurements are made in the air close to the animal’s nostrils, and measurements may not be affected by head position unlike sniffer method of Garnsworthy et al. (59). In a study with dairy cattle, a relatively strong correlation between CH_4_ measurements using the LMD with those determined in the RC (*r* = 0.80) was noted (62). The LMD can also strongly detect periods of high-enteric CH_4_ concentration and avoid misclassifying periods of low-enteric CH_4_ concentration (60). However, in a subsequent study, weak relationships (*r* = 0.22–0.28) between RC and LMD methods in CH_4_ measurements (61) have been reported. This technique allows measurements of CH_4_ in same animal repeatedly in their normal environments, while measurements are restricted during milking and feeding periods for the sniffer and GF techniques. However, the LMD system is labor intensive and meteorological factors, such as wind speed and direction, temperature, humidity, and atmospheric pressure, may influence the accuracy and precision of the measurements, with wind speed being a major factor for grazing studies and outdoor measurements (63). Like the sniffer technique, this technique also requires further improvements if LMD could be suitable for quantification of CH_4_ from large numbers of animals in normal management conditions for screening of animals on farms.

#### Methane Hood System

A novel method to quantify CH_4_ emissions during feeding has been designed recently by Troy et al. (64). This method can be used to measure CH_4_ output from individual animals in a group housed environment. In principle, this method is similar to GF system except that there is no requirement to provide extra feed supplements for enticement to visit an animal into the measurement area as required for GF system. Methane hood system measures CH_4_ concentrations in a hood designed to partially enclose the volume above a feed bin. Number of visits could be higher in methane hood than GF system, which may provide high accuracy in methane hood system. Troy et al. (64) compared this system with RC for measurement of CH_4_ emission employing nitrate as a CH_4_ mitigation option and found a comparable results between the RC method and this technique and detected significant treatment differences in CH_4_ production. Preliminary results suggest that this system could be a better alternative choice to quantify CH_4_ emissions from individual animals housed in a group in “natural” environments.

### Herd Scale Measurement Technique

#### Polytunnel Method

Polytunnel method requires a large tunnel made up of polyethylene fitted with end wall and large diameter port (43). As described by Lockyer and Jarvis (43), measure volume of air is continuously blown into and drawn from the large tunnel containing grazing animals, concentration of CH_4_ between the incoming and outgoing air is regularly measured, and temperature and humidity are monitored. Lockyer and Jarvis (43) and Lockyer (65) conducted two experiments using a polytunnel system of 4.3 m wide × 9.9 m long × 2.1 m height with an approximate volume of 66 m^3^ in which different numbers of sheep and calves were enclosed for up to 10 days. The recovery percentage of added CH_4_ in the tunnel was 104%. Average CH_4_ production was 13–14 and 74.5 g/day for sheep and calves, respectively. However, CH_4_ emissions decreased with increasing time of grazing, perhaps due to declining in available forage mass in the pasture as a result of very high stocking rates. Murray et al. (66) carried out two experiments to evaluate polytunnel system in comparison with the RC system for CH_4_ emissions from sheep. In both the system, the sheep were fed at maintenance levels of either fresh cut grass or dried grass pelleted diets. The results showed that CH_4_ production using the RC technique was greater (31.7 L/kg DM intake) than the tunnel technique (26.9 L/kg DM intake). The recoveries of the added CH_4_ in both the systems were similar (95.5–97.9% for tunnel versus 89.2–96.7% for RC). This system is suitable for measuring CH_4_ in semi-normal grazing conditions in individual or small group of animals. The operation of this method is simpler and portable, but there is difficulty in controlling temperature and humidity inside the tunnel.

#### Micrometeorological Techniques

During the last 10–15 years, a number of CH_4_ emission measurement techniques based on micrometeorological variables from whole farms, feedlots, and paddocks have been developed (67, 68). Micrometeorological methods involve measuring fluxes and concentrations of gases in the free atmosphere of a large area containing animals, and relating these fluxes and concentrations to calculate gas emissions from animals. Micrometeorological dispersion methods cannot measure emissions from individual animals as well as indoor housed animals. Furthermore, the scale of micrometeorological techniques makes their use difficult for testing mitigation options (69).

The micrometeorological methods involve measurements of CH_4_ concentration and wind speed, but the number of points of measurement and the assumption utilized to compute emission rates vary depending upon the methods. In the external tracer ratio technique, a tracer gas is released in the paddock or barn area, and the tracer gas and CH_4_ concentrations are measured in the surrounding areas (68). This category of methods also includes a mass balance technique in enclosed barns, where CH_4_ emissions are determined from ventilation rate and concentrations in inlet and outlet air (10). While it is relatively easy to estimate emission rates from mechanically ventilated closed barns, naturally ventilated buildings are problematic because of difficulties with measuring air exchange rates (10, 70). Air exchange rates in the naturally ventilated buildings depend upon the temperature gradient, temperature humidity index, and the wind speed. The release rates of gases may also depend upon outside environment, such as wind speed, humidity, and the other parameters (10).

A considerable development in micrometeorological techniques has improved CH_4_ measurement accuracy using inverse dispersion method (71). Inverse dispersion technique has been employed with success in many feedlot gas emission studies (69, 71, 72). This method has some advantages, such as non-interference of natural behaviors of animals and estimation of carbon footprint over large areas (73). However, there are also many limitations of inverse dispersion method, including wind conditions and the need for source homogeneity (73).

Emissions of CH_4_ from grazing animals are measured in field experiments using paddock-scale micrometeorological methods (74). The paddock-scale techniques analyze the patterns of transportation and dispersion of CH_4_ emitted from animals by the wind (74). Consequently, the CH_4_ emission rates are computed from measurements of wind speed, wind direction and characteristics of turbulent airflow, and CH_4_ concentrations in the direction and against the direction of wind (74). The paddock-scale methods estimate CH_4_ emissions using flux-gradient method, mass-budget approach, and gas dispersion models (9, 74). A comparison between RC and this method show similar CH_4_ yield, i.e., 30.1 versus 29.7 g/kg DM intake (75). Accuracy in measurement is dependent upon certain meteorological and landscape conditions, such as wind velocity and direction, topography of the land, and location of animals in the paddock (74). The micrometeorological methods are expensive and require sensitive instruments to analyze CH_4_ concentration (9, 11). Because several meteorological factors influence the accuracy of CH_4_ outputs, further developments and documentations for obtaining consistent results are needed, but the methods are valuable in evaluating CH_4_ emissions and carbon footprint in whole farm systems and interactions between animals and landscape.

### Indirect Measurements

#### *In vitro* Measurements

The *in vitro* rumen fermentation techniques have been extensively used for assessment of nutritive value of feeds for many years (76) and the techniques have been improved to simulate the rumen conditions. In this technique, feeds are fermented for long term [rumen simulation technique (77)] and short term [gas production methods (78)] under controlled laboratory conditions by rumen microbial activities. The volume of total gas production during incubation is determined and CH_4_ concentration in the gas is analyzed to obtain volume of *in vitro* CH_4_ production. With this system, the maximum level of total gas production and CH_4_ production can be determined, as well as the kinetics of gas production. Gas volumes are measured in different techniques (8) either directly by determining its volume at atmospheric pressure, e.g., Hohenheim gas production method or Menke’s method (78) and liquid displacement system (79) or by determining pressure changes due to accumulation of gas in a fixed volume container using a manometric device (80), a pressure transducer device with computerized (81) and manual (82) recordings, and a combination of pressure transducer and gas release device (83). Factors affecting the gas production in *in vitro* rumen fermentation system have been described in details by Rymer et al. (84). Recently, it has been shown that several other factors, such as bicarbonate concentrations in media and headspace gas composition (85), closed versus vented rumen batch culture system (86), and substrate dispersed in the medium versus kept in filter bags (87), influence the CH_4_ production in this technique. For diets containing different fiber concentrations and digestibility, CH_4_ production was close to that measured in RC method (88). Although several factors affect gas and CH_4_ production in the *in vitro* techniques, a fast screening of feedstuffs and additives for CH_4_ production is possible using these cost-effective simple techniques.

#### Modeling Enteric CH_4_ Production

Measurement of CH_4_ emissions in animals is difficult and labor intensive, and requires sophisticated and expensive equipments. Mathematical models predict CH_4_ emissions from ruminants without undertaking extensive and costly experiments. Therefore, prediction models are widely used for estimating national or global emissions from animals. The models used can be categorized as statistical models, which estimate CH_4_ production from nutrient intake directly [e.g., Ref. (2, 89)], or dynamic mechanistic models, which predict CH_4_ emissions using mathematical descriptions of rumen fermentation biology [e.g., COWPOLL model (90); MOLLY model (91)].

Mechanistic models (e.g., MOLLY and COWPOL) have advantages over the empirical statistical models in that CH_4_ mitigation technologies adopted at a farm or national level can be evaluated for their efficacy. Empirical models can evaluate the changes in CH_4_ emissions only in relation to changes in numbers of animals and feed intake. Diet-specific mechanistic models can more accurately predict CH_4_ emissions in ruminants (92). However, due to complexities of the mechanistic models, preparation of national inventory of CH_4_ estimates may not be straightforward. The Intergovernmental Panel on Climate Change (93) and Food and Agricultural Organization (1) publishes guidelines that are usually employed for official estimates of CH_4_ emissions in different countries. However, accuracy of these models to predict CH_4_ emissions has been challenged in different studies with cattle, buffaloes, sheep, and goats (2, 89, 94–96). The IPCC (93) developed methodologies to estimate enteric CH_4_ emissions with the use of CH_4_ conversion factor (Ym). However, Ym does not directly represent variations in CH_4_ emissions resulted from the ruminal fermentation characteristics affected by different carbohydrates, dietary nutrient composition, and feeding levels. Thus, the utility of Ym-based models in predicting enteric CH_4_ emissions and assessing the dietary CH_4_ mitigation strategies has been criticized (94). The low predictive ability of the Ym approach may invite substantial inaccuracy in preparation of enteric CH_4_ emission inventory (89, 94). Moreover, the IPCC and FAO models are generally developed based on the inputs from cattle. There was no model for predicting CH4 emission from buffaloes, goats, tropical cattle, and sheep. Recently, several statistical models have been developed for buffaloes, goats, sheep, and tropical cattle (Table 1). These newly fitted models performed better than the IPCC (93) and FAO (1) models as the recently developed equations had lower RMSPE values compared with these extant models (95). These new models should be considered for accurate preparation of enteric CH_4_ emission inventories for buffaloes, goats, sheep, and tropical cattle. For example, Patra and Lalhriatpuii (95) showed that the estimates of CH_4_ emission by goats were 5.23 and 5.15 kg/goat annually (actual CH_4_ production was 5.22 kg/goat/year) using the equations based on gross energy intake and digestible energy intake for goats, respectively. The estimate of CH_4_ emission using FAO (1) was 6.78 kg/goat/year, which was substantially greater than actual CH_4_ production. IPCC (93) suggested a CH_4_ emission factor of 5 kg/goat/year, which underestimated emissions. Similarly, based on the IPCC (93) tier II model, total enteric CH_4_ emission from buffaloes in India was estimated to be 4584 Gg/year in 2007 (5, 97). However, the estimate of enteric CH_4_ production from buffaloes using the equation based on DM intake (2, 89) was 4203 Gg/year, which was 8.3% lower than IPCC (93) model-based estimate (2, 89).

**Table 1:** **List of developed linear and non-linear statistical models used to predict CH_4_ production (MJ/day) from buffaloes, sheep, goats, and tropical cattle**.

Species	Equation: CH_4_ (MJ/day)	RMSE	*R*^2^	RMSPE%
Sheep (96)				
Eq. 1	= 0.208_(±0.040)_ + 0.049_(±0.0039)_ × GE intake (MJ/day)	0.24	0.86	22.7
Eq. 2	= 0.550_(±0.172)_ + 1.299_(±0.126)_ × DM intake (kg/day) − 0.266_(±0.053)_ × FL − 0.00093_(±0.00042)_ × NDF (g/kg)	0.22	0.92	22.4
Eq. 3	= −0.784_(±0.269)_ + 0.138_(±0.0084)_ × ME intake (MJ/day) − 0.378_(±0.062)_ × FL + 0.00294_(±0.00046)_ × OMDm − 1.943_(±0.381)_ × metabolizability	0.21	0.94	24.5
Eq. 4	= 5.699_(±1.94)_ − [5.699_(±1.94)_ − 0.133_(±0.047)_] × exp[−0.021_(±0.0071)_ × ME intake (MJ/day)]	0.14	0.91	20.7
IPCC (93)^a^	= 0.065 × GE intake (MJ/day)	–	–	23.1
FAO (1)^b^	= [(9.75 − 0.005 × DM digestibility g/kg)/100] × GE intake (MJ/day)	–	–	30.6
Goat (95)				
Eq. 5	= 0.242_(±0.073)_ + 0.0511_(±0.0073)_ × DE intake (kg/day)	0.31	0.83	30.3
Eq. 6	= −1.042_(±0.271)_ + 2.205_(±0.395)_ × NDF intake (kg/day) − 2.417_(±1.102)_ × EE intake (kg/day) + 1.456_(±0.323)_ × NFC intake (kg/day) + 0.0208_(±0.0039)_ × OMDm (g/kg) − 0.513_(±0.137)_ × FL	0.14	0.82	30.3
Eq. 7	= 0.885_(±0.154)_ + 0.809_(±0.0867)_ × DM intake (kg/day) − 0.397_(±0.0494)_ × FL + 0.0198_(±0.0022)_ × OMDm (g/kg) + 2.04_(±0.234)_ × ADF intake (kg/day) − 8.54_(±0.548)_ × EE intake (kg/day)	0.24	0.88	36.3
Eq. 8	= 1.721_(±0.151)_ × {1 − exp[−0.0721_(±0.0092)_ × ME intake (kg/day)]}	0.17	0.79	38.0
Buffalo (2, 89)				
Eq. 9	= 1.29_(±0.576)_ + 0.788_(±0.099)_ × DM intake (kg/day)	–	0.81	19.4
Eq. 10	= −0.436_(±0.665)_ + 0.678_(±0.184)_ × DM intake (kg/day) + 0.697_(±0.347)_ × NDF intake (kg/day)	–	0.85	16.1
Eq. 11	= 21.71_(±3.84)_ − [21.71_(±3.84)_ − 0.732_(±0.637)_] − exp[−0.0485_(±0.0094)_ × DM intake (kg/day)]	–	0.79	21.2
Cattle (94)				
Eq. 12	= 9.311_(±1.060)_ + 0.042_(±0.001)_ × GE intake + 0.094_(±0.014)_ × NDF − 0.381_(±0.092)_ × EE + 0.008_(±0.001)_ × BW + 1.621_(±0.119)_ × MF; for lactating cattle	2.59	–	15.6
Eq. 13	= 2.880_(±0.200)_ + 0.053_(±0.001)_ × GE intake − 0.190_(±0.049)_ × EE; for non-lactating cattle	1.29	–	14.4
Eq. 14	= 1.487_(±0.318)_ + 0.046_(±0.001)_ × GE intake + 0.032_(±0.005)_ × NDF + 0.006_(±0.0007)_ × BW; For heifer cattle	1.23	–	18.6
Eq. 15	= 0.221_(±0.151)_ + 0.048_(±0.001)_ × GE intake + 0.005_(±0.0005)_ × BW; for steer	0.92	–	15.1
Tropical cattle (98)			
Eq. 16	= 1.29_(±0.906)_ + 0.878_(±0.125)_ × DM intake	5.49	0.70	31.0
Eq. 17	= 0.910_(±0.746)_ + 1.472_(±0.154)_ × DM intake − 1.388_(±0.451)_ × FL − 0.669_(±0.338)_ × ADF intake	4.22	0.84	22.2
Eq. 18	= 71.47_(±22.14)_ × [1 − exp(−0.0156_(±0.0051)_ × DM intake)]	3.56	0.83	30.3

*^a^IPCC (93) had RMSPE% of 52.4, 27.2, 18.6–30.5, and 32.5 for the database of goat, buffalo, cattle, and tropical cattle, respectively*.

*^b^FAO (1) had RMSPE% of 51.0 and 19.2–29.7% for the database of goat and cattle, respectively*.

*GE, gross energy; DE, digestible energy; DM, dry matter; NDF, neutral detergent fiber; FL, feeding level; ADF, acid detergent fiber; ME, metabolizable energy; EE, ether extract; NFC, non-fiber carbohydrate; OMDm, organic matter digestibility at maintenance level of feed intake; MF, milk fat (%); BW, body weight (kg); RMSE, root means square error; RMSPE, root mean square prediction error*.

Static empirical models have advantages in that they are usually based on a small number of variables (e.g., DM intake, feeding level, dietary lipid%, dietary fiber%, etc.), they can be performed in a simple spreadsheet, and they are transparent and can be easily tested on a variety of datasets (35). The disadvantage of static models is that they do not rely on an understanding of the biology and biochemistry of methanogenesis in the rumen, and they are, therefore, of limited use to study new CH_4_ mitigation strategies. Static empirical models are also restricted in predicting CH_4_ production beyond the data used for their development. The national GHG inventory in many countries uses a static empirical model to estimate emissions of CH_4_ from ruminant livestock. Nevertheless, prediction models are the strong base for estimating national or global emissions from animals.

#### Proxy Measures of CH_4_ Emissions

A considerable research effort has been directed toward development of proxy measures for predicting enteric CH_4_ production from composition of milk and feces. Several studies examined the concentrations of certain fatty acids in milk as predictors of CH_4_ production from dairy animals (99, 100). The assumption in this approach is that specific fatty acids in milk or feces are correlated with the composition of feeds or the amount of rumen methanogenic archaea, which would influence CH_4_ emissions in the rumen (100). Williams et al. (101), however, observed weak correlations between CH_4_ production and the concentrations of specific fatty acids in milk fat. Few studies (100, 102) indicated some correlations between milk fatty acid profiles and CH_4_ emissions. Milk mid-infrared spectra (influenced by milk fatty acid composition) measured using FTIR analysis apparatus could directly better predict CH4 emission (103) compared with fatty acid composition.

The use of archeol (2,3-diphytanyl-O-sn-glycerol), which is a membrane lipid ubiquitous in methanogens has been explored as a potential molecular proxy for methanogenesis in cattle (104). A significant correlation between fecal archeol concentration and CH_4_ production measured using the SF_6_ and RC technique in cattle fed either a grass- or concentrate-based diet was observed, but relationships between individual measurements within dietary treatments were weak to moderate, possibly due to selective retention of archaea in the rumen and degradation of the archeol during gut transit, differences in the CH_4_ producing capability per cell and post excretion (104, 105). These methods could be useful to predict the individual CH_4_ production to identify low-CH_4_-emitting animals, but further research is needed to improve the predictability of CH_4_ emissions using these proxy methods.

### Other Potential Technology

#### Intra-Ruminal Gas Sensor

An intra-ruminal device, which measures the concentrations of CH_4_ and CO_2_ dissolved in rumen fluid, but does not measure flux (emission), has recently been fabricated (106). The rumen environmental conditions may be specifically unfavorable for an electronic device, which may cause corrosion of electrical circuits. In addition, the dissolved gases in rumen fluid must permeate quickly through the membrane of the intra-ruminal device in order to dynamically analyze the concentrations of gases (35). Information on internal rumen pressure, rumen size, and eructation pattern can be integrated to estimate the gas production rates (11). Thus, further research would be required to develop an approach to measure CH_4_ production from individual animals from the *in situ* measurements of gas concentrations in the rumen. The measurement of CO_2_ and CH_4_ concentrations in rumen and breath (respiratory and eructated) at the same time would be advantageous to assess the feasibility of using CO_2_ as a tracer gas and this could guide to the use of low-cost handheld systems to estimate CH_4_ production (11).

## Dietary Strategies to Mitigate CH_4_ Emissions

**Figure 2:**
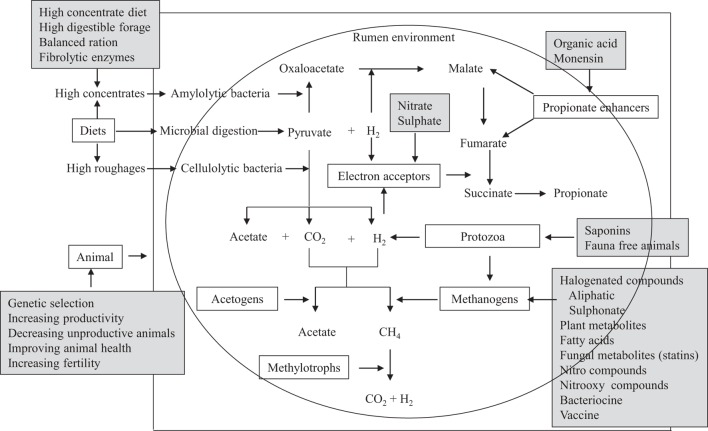
**A schematic presentation of the potential targets of decreasing CH_4_ emissions from ruminants**. Boxes without dark could be the targets for suppressing CH_4_ emissions and boxes with dark shade are the options that have been studied *in vitro* or *in vivo* to decrease CH_4_ production [adapted with modification from Patra (5, 97)].

Several mitigation options and strategies have been explored, which involve intervention at the animal level, dietary composition of animals, modulation of rumen fermentation, and inhibition of methanogenic archaea (Figure 2). Methanogen-specific inhibitors could be potentially effective mitigation agents if they utilize the evolutionary distinctiveness of methanogenic archaea (35). Archaea are evolutionarily distinct from other rumen microorganisms (bacteria, protozoa, fungi, and viruses), and all methanogenic archaea share a similar biochemical pathway of methanogenesis (107). Therefore, the inhibitors of this methanogenesis pathway may distinctively inhibit only methanogens without directly influencing other beneficial microorganisms in the rumen (35, 108). Several reviews on CH_4_ mitigation strategies and options have been published recently (3, 6, 97). In this section, further recent advances in dietary CH_4_ mitigation technologies are described here.

### Lipid Supplementation

Several studies have confirmed that addition of fats or fatty acid to the diets of ruminants can decrease enteric CH_4_ emissions [e.g., Ref. (2, 89, 101, 109)]. Each percentage increase in supplemental dietary fat decreases CH_4_ emission by 4.30% (109). Fat concentrations of up to 6% of diet DM may also increase milk production and lower enteric CH_4_ emissions appreciably (15%) in cattle (109), which is a win–win situation. Fat concentration beyond this concentration may decrease production efficiency due to adverse effect on rumen fermentation. Among fatty acids, C12:0, C18:3, and poly unsaturated fatty acids have more marked CH_4_ suppressing effects, whereas saturated long-chain fatty acids are less effective for decreasing CH_4_ production in cattle (109, 110). The by-products containing high concentration of lipids (e.g., brewers grains, grape marc, hominy meal, etc.) appear to be promising to mitigate CH_4_ emissions cost-effectively (101). Grainger et al. (24) showed that supplementary feeding of whole cottonseed to dairy cows could cause a substantial decrease in CH_4_ emissions without adversely affecting milk production. Moate et al. (111) compared brewers grains, cold-pressed canola, and hominy meal for their CH_4_ mitigation potential and found that all three by-products could substantially reduce enteric CH_4_ emissions from dairy cows. The feeding of red grape marc to dairy cows decreased CH_4_ emissions and CH_4_ yield by 20% (32). In addition to the presence of fat, grape marc also contains many secondary compounds such as tannins, p-coumaric acid, and resveratrol, which may also inhibit enteric methanogenesis (32).

### Plant Secondary Compounds

Several plant secondary metabolites present in forages and plant extracts have been identified to be potential for CH_4_ inhibition in the rumen (108). Many forage plants rich in tannins and saponins have shown to be promising to reduce CH_4_ production in ruminants (112, 113). Tannins may decrease CH_4_ directly by inhibiting methanogenic bacteria, and indirectly by decreasing hydrogen production as a result of decreased fiber digestion and protozoal population in the rumen (108).

A recent investigation with different forage brassicas have shown that most of the brassicas species fed to sheep resulted in similar CH_4_ yields, However, swedes and forage rape significantly decreased CH_4_ yield (~20%) compared with ryegrass and other brassicas, such as turnips and kale (114). Although the mechanism of CH_4_ inhibition is not clearly known, these forages may contain S-containing plant metabolites, which may be responsible for inhibition of CH_4_ production (108). Additional research is required to examine the CH_4_ mitigation effects of these forage brassicas fed to ruminants for long time.

The studies on flavonoid compounds on rumen methanogenesis are limited although other metabolites, such as tannins, saponins, and essential oils, have extensively been studied (108). A recent study reported that inclusion of flavone, myricetin, naringin, rutin, quercetin, and kaempferol significantly decreased *in vitro* CH_4_ production from 8.6 to 5.7, 4.9, 6.3, 7.2, 6.2, and 5.3 mL/g DM, respectively. The inhibitory activities of flavonoids used in this experiment toward methanogenesis were in the following descending order as follows: myricetin ≥ kaempferol ≥ flavone > quercetin ≥ naringin > rutin ≥ catechin (115). Catechin decreased CH_4_ production both *in vitro* (116) and *in vivo* (117). Catechin causes direct inhibition of methanogens (115) as well as may act as hydrogen sinks during degradation by rumen microbes via cleavage of ring structures and reductive dehydroxylation reactions (116).

### Nitrate Supplementation

Nitrate decreases methanogenesis acting as electron sinks and directly inhibiting the methanogens (85, 118). Use of nitrate has two advantages – (1) it decreases CH_4_ production and (2) it provides ammonia to the rumen microbial growth resulting in decreased dietary protein requirement. Nitrates as a dietary supplement fed to dairy cows in low nitrogen containing diet have been shown to reduce CH_4_ emissions (119). However, dietary nitrate supplementation may increase the risk of nitrite toxicity, particularly for the forages containing high concentrations of nitrate and crude protein.

### Halogenated Compounds

Halogenated compounds had been investigated as inhibitors of CH_4_ production in the rumen over 40 years ago (5, 97). Some marine plants, such as the red seaweed, algae, and fungi, contain bromoform and other halogenated compounds at high concentrations (120), which have been exploited recently to inhibit CH_4_ production. In a recent *in vitro* experiment, red seaweed, *Asparagopsis taxiformis*, was shown to reduce CH_4_ production by 99% when it was used at 2% of organic matter substrate (121). Thus, the supplementation of ruminant diets with red sea weed may offer a natural means of CH_4_ mitigation. *In vivo* experiments are required to decide optimum dose levels and to study the toxic effect of the weed, if any.

### Nitrooxy Compounds

Novel inhibitors 3-nitrooxypropanol (NOP) and ethyl-3NOP have been shown to have specific anti-methanogenic properties. NOP interferes with the methyl-coenzyme M reductase of the methanogens, which is the last step in the formation of CH_4_, thus lowering CH_4_ production and inhibition of the growth of methanogens (122, 123). Reynolds et al. (124) noted a 7–10% lower in CH_4_ production when NOP was administered directly into the rumen of cattle through a rumen cannula at a daily dose of 0.50 or 2.5 g per cow (i.e., 25 or 125 mg/kg DM). Digestibility decreased at high dose in this study. However, in subsequent studies, Haisan et al. (125) noted 60% decrease in CH_4_ emission in cattle fed NOP at a dose of 2.5 g/day mixing with the feed to ensure continuous intake of this compound throughout the day. Feeding of NOP at 40–80 mg/kg diet in dairy cattle was also associated with decreased CH_4_ production by 30% (126). In beef cattle diet also, 3NOP added to the diets at 2.0 g/day decreased CH_4_ yield by 59% up to 112 days of experiment without much affecting feed intake, nutrient digestibility, and total volatile fatty acid (VFA) concentrations (127). In this study, the numbers of methanogens were reduced, but protozoal numbers were increased by 3NOP. These studies suggested that 3NOP needs to be continuously supplied to the diet to get optimal inhibitor effect of CH_4_ production. The results have been confirmed in other study (128) where CH_4_ yield was lowered by 37% due to feeding of 3NOP at 2.5 g/day in dairy cow. In sheep also, 3NOP at 0.5 g/animal per day decreased CH_4_ production by 29% without adversely affecting digestion and rumen fermentation (123). It appears that 3NOP is a CH_4_ inhibitor, which has potential for reducing carbon footprint of livestock products without affecting nutrient utilization and performance in ruminants.

### Fungal Metabolites

Lovastatin is a secondary metabolite of idiophase of the fungi, which inhibits the key enzyme of cholesterol biosynthesis, such as enzyme 3-hydroxy-3-methyl glutaryl coenzyme A (HMG-CoA) reductase (129). *Aspergillus terreus* fungi fermented rice straw extract containing lovastatin significantly reduced CH_4_ production and number of methanogens, but increased few fiber degrading bacteria (129). Ether-linked long-chain isoprenoid alcohol is a central component in archeal cell membrane lipid, which is produced from a key precursor, mevalonate. Mevalonate is synthesized by reduction of HMG-CoA catalyzed by HMG-CoA reductase and it is an intermediate rate limiting reaction in synthesis of cholesterol in human (108). Lovastatin is an inhibitor of HMG-CoA reductase, which inhibits isoprenoid alcohol synthesis, and consequently archeal cell membrane formation and growth of methanogens could be retarded (129). Strains of saprophytic fungi *Mortierella wolfii* were also promising as an inhibitor of methanogenesis without also reducing overall fermentation (130). In another study in sheep fed Monascus-fermented rice, CH_4_ emission was decreased by 30% by fungal metabolites (possibly pravastatin and mevastatin) produced by *Monascus* spp., which was associated with lower ruminal acetate to propionate ratio and decreased numbers of methanogens in the rumen (131).

### Microalgae

An effort has been started to screen microalgae to inhibit rumen methanogenesis. Machado et al. (121) screened several marine microalgae *in vitro* and reported that *Asparagopsis* not only strongly decreased CH_4_ production by 99% at a dose of as low as 2% of total substrate organic matter, but also decreased the production of VFA. *Oedogonium* was less effective with doses ≥50% OM significantly decreasing the production of CH_4_. A combination of *Asparagopsis* (2% OM) and *Oedogonium* (25 and 50% OM) continued to suppress the production of CH_4_, independent of the inclusion rate of *Oedogonium*. The brown algae (*Cystoseira trinodis* and *Dictyota bartayresii*) were also identified as a potent CH_4_-inhibiting agent *in vitro* (132). One algal meal containing 20% docosahexaenoic acid fed to cows up to 375 g/cow per day of algal meal corresponding up to 75 g of docosahexaenoic acid/cow per day did not affect CH_4_ production *in vivo*, which was probably due to the low concentration of this fatty acid to inhibit CH_4_ production (133).

### Use of Combination of CH_4_ Inhibitors

A number of CH_4_ inhibitors have been frequently evaluated, primarily individually, to lower CH_4_ production in ruminants. However, they generally exert detrimental effects on digestion of feeds and rumen fermentation when they are added at high concentration in order to obtain substantial effect on CH_4_ inhibition (5, 97, 134, 135). Some of these inhibitors also cause toxicity to animals when used at large doses (97). These adverse effects on rumen fermentation and toxicity problems to animals can be overcome at low doses, but substantial effect on inhibition to methanogenesis is not noted at low doses. Combinations of inhibitors with complementary modes of actions may synergistically or additively lower CH_4_ emission without exerting any detrimental effects on digestion of feeds or rumen fermentation at low doses (134). Indeed, this hypothesis has been confirmed in some studies (134–138). In a study, a binary combination of nitrate and quillaja saponin inhibited methanogenesis additively in an *in vitro* rumen culture [by 32% at 5 mM nitrate and 0.6 g/L saponins, and by 58% at 10 mM nitrate and 1.2 g/L saponins (134)]. Binary combination of nitrate and saponins might act additively to decrease methanogenesis in a multipronged manner: (1) saponins inhibit rumen protozoa, lowering hydrogen production by protozoa and reducing the abundance of protozoa-associated methanogens (139), (2) nitrate functions as a strong electron sink that outcompetes CO_2_ for electrons, and (3) nitrite, the first intermediate of nitrate reduction, exerts direct toxicity to methanogens. However, binary combination of high doses of nitrate and saponins decreased fiber degradability (85). Garlic oil is directly inhibitory to rumen methanogens acting through impairment of lipid synthesis (140). Combination of saponin + nitrate + sulfate, garlic oil + nitrate, and garlic oil + nitrate + saponin resulted in additive effects on methanogenesis (118, 136, 140). Hops extract (*Humulus lupulus*; containing β- and α-acids) and yucca saponin decreased CH_4_ in an additive manner when applied in combination (138). Additive effects of combinations of CH_4_ inhibitor have been tested a little *in vivo*. A recent *in vivo* study demonstrated that nitrate (3% calcium nitrate; 22% reduction) and linseed oil (4% of the diet; 17% reduction) in combination (32% reduction) for decreased methanogenesis additively in cows without altering diet digestibility (141). It appear that combinations of CH_4_ inhibitors with complementary mechanism of actions could be useful technology to substantially decrease CH_4_ production without adverse effect on nutrient utilization, but more *in vivo* research is required to identify the suitable dose combinations for practical on farm application.

## Conclusion and Future Research Challenges

Many existing methods have been employed to measure CH_4_ production with different purposes, such as nutritional evaluation of feeds and feed additives and screening of animals for genetic selection, and have been investigated to improve accuracy of measurements. Many new techniques are being developed to overcome the constraints of the existing methods. However, no method is suitable in all conditions for reliable measurement of CH_4_ emissions. Every method has its advantages and limitations, and a method is useful in particular conditions of a study for CH_4_ measurement and mitigation. Most consistent RC method is only a “gold standard” when this is used with adequate rigor and technical expertise. The SF_6_ method can be employed with lesser effect on animal behavior and has a higher throughput relative to time and cost. With recent recommendations for the technique, the SF_6_ method can be used with a high level of precision in grazing animal studies for long time.

The short-term measurement techniques have advantages as it is relatively cheaper, simpler, and mobile compared to other techniques, such as SF_6_ or RC. All short-term measurements invite variations at several points, such as measurement time relative to feed intake and level of activity before measurement (45). The higher within and between animal-to-animal and day-to-day variations in CH_4_ emissions would require more number of animals and measurement days to obtain significant differences of CH_4_ emissions between treatments using short-term measurement techniques. Daily CH_4_ emission patterns of ruminants generally show a diurnal pattern in relation to the feeding time and level of feed intake (58, 142). The diurnal profiles associated with feed intake exhibit a continuous rise to a peak followed by a period of a linear decline. Therefore, for short-term measurements of CH_4_ emissions, such as sniffer method (59) taken twice during the daily feeding and milking time, estimation of daily CH_4_ production based on measurement of short-term emission rates will be diet dependent (45). For systems such as GF and methane hood where short-term emission rates are measured throughout 24 h, daily CH_4_ emission may be estimated without scaling up (45). For systems such as for PACs and LMD where small numbers of measurements for emission rates are taken and feed intake information of an animal is not known, scaling up to daily CH_4_ emission is not currently possible (45). Short-term measures of CH_4_ emission may strongly correlate with daily CH_4_ emission depending on the time after feeding at which the short-term measurements are made. Thus, short-term measuring techniques can be applied to very large numbers of animals under normal management conditions to collect information on low CH_4_-producing animals required for genetic selection purpose. Further research efforts are needed to improve the accuracy of all the new methods.

Several new CH_4_ mitigation technologies have been explored, but only a few of them are practical and cost-effective, which can be adopted on farms to achieve substantial mitigation of total CH_4_ emissions. A combination of different CH_4_ mitigation strategies should be adopted in farm levels to substantially decrease CH_4_ emission from ruminants. The CH_4_ mitigation options that show both nutritional and environmental advantages would likely to be better adopted by the farmers. For example, fat supplementation could decrease CH_4_ production as well as improve productivity of animals. Similarly, nitrate supplementation could reduce the expensive protein meals in diets. If some mitigation technologies could be employed to improving the nutritional values of forages, they have immense practical importance in tropical feeding situations. For example, if saprophyte fungi, which produce metabolites against methanogens, could be grown on low-quality forages, such as straws, this technology would be feasible for decreasing CH_4_ production in ruminants. Toxicities and residues in milk and meat associated with CH_4_ inhibitors must be assessed with long-term trials in animals. Many fungi, for example, may produce toxic metabolites in some growth conditions, which must be avoided for practical feeding of animals (143). Nitrate supplementation may cause toxicities in animals if it is not used in proper doses with relation to the nitrate content in forages. Future mitigation research should focus for the developments of both the short-term strategies, such as dietary strategies and animal management as well as long-term strategies focusing on plant and animal breeding, and rumen microbial modulation in order to profitably decrease carbon footprint and strengthen the future sustainable and environment friendly livestock production systems.

## Author Contributions

The author confirms being the sole contributor of this work and approved it for publication.

## Conflict of Interest Statement

The author declares that the research was conducted in the absence of any commercial or financial relationships that could be construed as a potential conflict of interest.
